# Oestrogen replacement combined with resistance exercise in older women with knee osteoarthritis: a randomised, double-blind, placebo-controlled clinical trial

**DOI:** 10.1093/ageing/afaf224

**Published:** 2025-08-07

**Authors:** Tomohiro Mitoma, Hikaru Ooba, Kasumi Takahashi, Tsunemasa Kondo, Tomohiro Ikeda, Yoko Sakamoto, Toshiharu Mitsuhashi, Jota Maki

**Affiliations:** Medical Development Field, Center for Innovative Clinical Medicine, Okayama University, Okayama, Japan; Obstetrics and Gynecology, Chugoku Central Hospital, Fukuyama, Japan; Medical Development Field, Center for Innovative Clinical Medicine, Okayama University, Okayama, Japan; Obstetrics and Gynecology, Ochiai Hospital, Maniwa, Okayama, Japan; Obstetrics and Gynecology, Ochiai Hospital, Maniwa, Okayama, Japan; Rehabilitation Medicine, Okayama University Hospital, Okayama, Japan; Medical Development Field, Center for Innovative Clinical Medicine, Okayama University, Okayama, Japan; Medical Development Field, Center for Innovative Clinical Medicine, Okayama University, Okayama, Japan; Medical Development Field, Center for Innovative Clinical Medicine, Okayama University, Okayama, Japan

**Keywords:** oestrogen replacement therapy, muscle resistance exercise, knee osteoarthritis, physical performance, randomised controlled trial, older people

## Abstract

**Background:**

Interventions targeting physical function decline in older women with knee osteoarthritis (KOA) are vital for healthy ageing. The additive benefits of combining oestrogen replacement therapy (ERT) with resistance exercise remain unclear.

**Objective:**

To evaluate the additive effect of low-dose ERT on physical performance when combined with a muscle resistance exercise programme (MREP) in older women with KOA.

**Design:**

This is a placebo-controlled, double-blind, randomised clinical trial.

**Subjects:**

The subjects were community-dwelling women aged ≥65 years with chronic knee pain and KOA diagnosis.

**Methods:**

Participants completed a 3-month MREP and were randomised to receive daily low-dose transdermal ERT (oestradiol 0.54 mg/day) or placebo. Outcomes were assessed at baseline, postintervention and 12 months later. The primary outcome was change in 30-second chair stand test (CS-30) score. Secondary outcomes included muscle mass, knee extension strength, walking performance, metabolic indicators, knee pain scale and 12-item short-form health survey (SF-12). Between-group differences in CS-30 changes were analysed using a linear regression model based on the intention-to-treat principle.

**Results:**

Among 168 individuals screened, 75 participants (mean age 73.8 years, SD 5.8) were enrolled and randomised into an ERT group (*n* = 37) or a placebo group (*n* = 38). Baseline CS-30 scores were 14.81 (SD 3.95) in the ERT group and 15.58 (SD 3.48) in the placebo group. At 3 months, mean changes were 2.59 (SD 2.58) and 1.79 (SD 2.28) repetitions, respectively. The primary analysis showed no statistically significant between-group difference [regression coefficient: 0.81 (95% CI: −0.31, 1.92); *P* = .16]. *Post hoc* subgroup and sensitivity analyses suggested that benefits may exist among early-stage KOA participants. SF-12 mental health scores also improved significantly in the ERT group. No serious adverse events occurred.

**Conclusions:**

ERT did not confer significant additive benefits to resistance exercise overall but may improve outcomes in early-stage KOA and mental health domains. These exploratory findings warrant further investigation.

## Key Points

- First RCT assessing oestrogen replacement therapy (ERT) combined with resistance exercise for physical function in older women with knee osteoarthritis (KOA).
- ERT showed no significant benefit overall but might improve outcomes under early-stage KOA or good adherence.
- ERT may have improved mental health scores, suggesting additional psychological benefits beyond physical function.
- Targeting early-stage KOA with ERT and exercise may optimise physical and mental health outcomes.
- Findings support further research into personalised interventions for older adults with KOA.

## Introduction

In the contemporary era of an accelerated super-aged society, joint diseases contribute to a decline in physical function and significantly reduce healthy life expectancy [1–3]. The prevalence of weight gain among older adults has emerged as a significant public health concern [4], accompanied by a rise in sarcopenic obesity, characterised by reduced muscle mass [5, 6]. Weight gain and reduced muscle mass are associated with the onset of joint disorders and frailty [7, 8]. Knee osteoarthritis (KOA) is a prevalent condition among postmenopausal women, with its incidence increasing significantly with advancing age [9, 10]. Knee pain negatively affects physical function by reducing daily activity levels, muscle mass and physical fitness while also hindering social interactions and contributing to isolation and loneliness [11, 12]. Moreover, joint diseases increase the number of individuals requiring long-term care, raise medical expenses and impose a substantial economic burden on society. Addressing this issue requires a comprehensive approach to extending healthy life expectancy through interventions targeting physical function decline due to knee pain and early preventive measures for sarcopenia [13].

The Osteoarthritis Research Society International (OARSI) guidelines for nonsurgical treatment recommend strength training exercises, land-based exercises, aquatic exercises, weight management, self-management and patient education as core interventions [14]. In recent years, numerous clinical trials on exercise therapy for knee pain have been conducted, demonstrating that resistance training effectively reduces knee pain, enhances lower-limb strength and contributes to improved physical performance. However, resistance training faces the challenge of adherence [15–17], highlighting the need for more efficient implementation over shorter durations [15]. Consequently, pharmacological treatments combined with exercise therapy to improve joint pain and physical performance in osteoarthritis have become a key research priority.

Oestrogen levels decrease markedly during menopause and are strongly associated with an increased incidence of cardiovascular disease and osteoporosis in postmenopausal women [18, 19]. Oestrogen receptors are present in joint cartilage, muscles and tendons, where oestrogen helps maintain their function [20–23]. Oestrogen has been reported to play a crucial role in preventing joint pain and supporting the development and regeneration of skeletal muscles unique to women [22, 24]. Studies on the effects of hormone replacement therapy (HRT) on muscle mass and strength around menopause indicate no significant impact of oestrogen on muscle mass [25–27], whereas its role in increasing muscle strength remains uncertain [28]. A significant challenge is the substantial bias observed in over half of clinical studies [28]. Resistance exercise is essential for improving muscle strength and physical performance; however, few studies have investigated the effects of muscle resistance exercise combined with HRT in postmenopausal women [29]. Furthermore, research on the relationship between oestrogen and muscle mass, as well as its association with physical performance, has predominantly targeted healthy women in the early postmenopausal period. Intervention studies focusing on women in their 70s–80s, where the impact of muscle weakness due to joint diseases becomes significantly evident, remain limited.

The aim of this study was to investigate whether transdermal oestrogen supplementation enhances the effects of muscle resistance exercise on physical performance in older women with KOA and to evaluate the sustained effects of this intervention. Additionally, it sought to provide new evidence regarding the efficacy of pharmacological treatments and maintenance programmes for older women at risk of physical function decline due to joint pain.

## Method

### Study design

The EPOK trial is a multicentre, placebo-controlled, double-blind, randomised clinical trial designed to evaluate the additive effects of oestrogen replacement therapy (ERT) combined with resistance exercise in older women with KOA. The study was conducted in Maniwa City, Okayama Prefecture, Japan, with Okayama University Hospital and Ochiai Hospital serving as registered and implementing research institutions. It was conducted in collaboration with the Maniwa City Community Comprehensive Support Centre, a local government agency, and the NPO Maniwa Agri-Garden Project. The trial protocol has been previously described [30]. The study adhered to the Standard Protocol Items: Recommendations for Interventional Trials 2013 guideline and Consolidated Standards of Reporting Trials.

### Participants

Participants were recruited through local advertisements, public seminars and social support systems. Those who expressed interest were invited to attend information sessions held by investigators in various local districts and senior community groups. Eligibility screening was conducted via interviews using a medical history and exclusion criteria checklist. Individuals who met the initial criteria and showed no clear exclusion factors were referred for a detailed medical assessment. Among those who underwent medical assessment, individuals who satisfied the eligibility criteria were enrolled as participants. Eligible participants were women aged 65–90 years who were independently ambulatory, had experienced knee pain for at least 3 months and had radiographic evidence of KOA classified as Kellgren–Lawrence Grade 1 or higher [31]. Given that exercise therapy for knee pain is applicable across the full spectrum of KOA severity [14], we included participants with a broad range of disease stages, from early-stage KOA (KL Grade 1) to those who had undergone total knee arthroplasty (TKA). This decision was also supported by the rationale that oestrogen may exert beneficial effects not only on intra-articular structures but also on periarticular muscles [21, 22]. Participants with contraindications to resistance exercise were excluded. Individuals were excluded if they (i) had been treated for conditions within the past 2 years that prevented safe participation in resistance exercise interventions; (ii) had a history of gynaecological disorders or breast cancer within the past 5 years contraindicating hormone therapy; (iii) had a history of cardiovascular disease or stroke within the past 2 years; (iv) had experienced fractures of the upper or lower limbs within the past 2 years; (v) had prolonged immobility; (vi) had anorexia, renal impairment or severe liver dysfunction; (vii) had uncontrolled hypertension; or (viii) had cognitive impairment. Full eligibility and exclusion criteria are shown in Appendix 1 in the supplementary data. All individual representatives provided written informed consent. No financial compensation was provided.

### Randomisation and blinding

Participants were randomly assigned to one of the two groups in a 1:1 ratio by an allocator to either the ERT or placebo group. Randomisation was stratified according to age, physical activity level and knee extension strength (KES). A computer-generated randomisation schedule created using the randMS® mobile application (developed by Shinji Maeda with Filemaker® and Filemaker Pro Advanced®, ©2019 kazenoan) and incorporating two or more block sizes ensured allocation concealment. The allocator sent participant numbers and allocation results to an unblinded pharmacist. The unblinded pharmacist prepared the study gels to maintain blinding integrity among participants and other trial staff members. Participants, investigators and analysis personnel were blinded to the group assignments. During the analysis, the two groups were labelled as Group A and Group B, and blinding was only lifted by the principal investigator after the analysis was completed. The visits to medical institutions from recruitment to allocation, as well as the flowchart, are presented in Appendices 2 and 3 in the supplementary data.

### Intervention

This study included two simultaneous interventions. The first involved the daily application of transdermal gel for 3 months. Participants applied either oestrogen gel (l’estrogel 0.06%®; 0.54-mg oestradiol per application; Fuji Pharma, Tokyo, Japan) or a visually identical placebo gel once daily to the anterior thigh. The placebo gel replicated the base composition, appearance and bottle shape of the oestrogen gel. Participants received instructions on correct gel application before the intervention. Medication adherence and potential side effects were monitored by measuring the mass of the gel containers both 4 weeks after the start and immediately after the end of the intervention, with additional guidance provided as needed.

The second intervention was a structured muscle resistance exercise programme (MREP). All participants received instructions on the MREP along with educational materials on the benefits of lower-limb exercises for KOA and the importance of adherence to the programme [14, 32]. The MREP included one weekly group session and two home sessions. Group sessions (12 in total) were held at community centres and led by instructors trained by physiotherapists. These sessions were designed based on the OARSI guidelines [14, 33], focusing on the four major lower-limb muscle groups (quadriceps, calf muscles, hamstrings and gluteal muscles), as well as abdominal and back muscles. Home sessions, scheduled twice weekly, replicated the group muscle resistance exercise content (25 minutes). Participants tracked their adherence on a calendar and rated their exercise completion. Instructors reviewed the calendars every 4 weeks and provided feedback. Analgesic agents, including nonsteroidal anti-inflammatory gels, were used as needed. Participants maintained normal daily activities, such as walking and stretching, but were instructed to avoid new invasive knee treatments, including surgery and fluid injections.

### Outcomes

All primary and secondary outcomes were measured at baseline and 3 and 12 months. The primary outcome was the change from baseline in physical performance, assessed using the 30-second chair-stand test (CS-30), a validated measure of lower-limb physical performance [34]. Participants used a 40-cm armless folding chair, sat upright with feet shoulder-width apart, arms crossed and completed as many full stands as possible in 30 seconds.

Secondary outcomes included the assessment of the daily average knee pain over the past week using the Visual Analog Scale (VRS) and functionality using the Short Form-12 (SF-12) [35]. Physical performance was further evaluated using the 5-m walking time and the timed up-and-go test (TUGT). Muscle strength was measured through grip strength, using a digital handgrip dynamometer (Takei-kiki Corp., T.K.K5401), and KES, using a handheld dynamometer with a fixing belt (Anima Corp., Mu-Tas F-2). Body composition and muscle mass were assessed using bioelectrical impedance analysis with Tanita MC-780AN and Tanita Zaritz BM-220 instruments (Tanita Corp., Tokyo, Japan). Blood markers assessed included albumin, creatinine, bilirubin, alanine aminotransferase, aspartate aminotransferase, gamma-glutamyltransferase, C-reactive protein, low-density lipoprotein, high-density lipoprotein, total cholesterol, triglycerides, oestradiol, insulin-like growth factor-1, calcium, 25-hydroxyvitamin D, haemoglobin A1c, haemoglobin, haematocrit and white blood cell and platelet counts.

### Adverse effects

Adverse effects were monitored at three time points—4 weeks after initiation, at the end of the intervention and 9 months later—with all events and complications systematically recorded throughout the follow-up period (Appendix 3 in the supplementary data). A 24-hour contact system was established at the study site to facilitate communication and provide medical consultations regarding adverse events. In the event of an adverse effect, the principal investigator conducted a thorough evaluation to assess the feasibility of continued participation. Potential adverse effects associated with ERT—including abnormal uterine bleeding, breast tenderness, vaginal discharge and local reactions at the gel application site—were prespecified in the study protocol, along with predefined management strategies. Symptoms suggestive of adverse reactions were listed in advance, with specific responses, such as drug discontinuation or medical referral, clearly outlined. Serious adverse events were explicitly defined and managed according to protocol. Post-study follow-up specifically targeted symptoms considered relevant to the safety of oestrogen therapy.

### Statistical analysis

Previous studies have demonstrated that oestrogen supplementation moderately improves lower-limb strength (5%–17%) and enhances joint function in osteoarthritis [24, 29]. Resistance exercise has been reported to increase CS-30 scores by 2.68 repetitions [95% confidence interval (CI): 1.90–3.47] [36]. The average CS-30 score in patients with KOA is 9.2 repetitions [37]. Assuming that ERT is expected to increase the CS-30 score by 13%, the sample size was determined to detect a 1.2-fold improvement in the primary outcome, as measured by the increased CS-30 score. Sample size calculations were based on prior research and the expected increase in repetitions attributed to MREP [28, 29, 36]. A sample size of 36 participants per group was calculated to achieve 80% power for a one-sided test, with a significance level of 5% and an effect size (Cohen’s *d*) of 0.6. To account for a potential dropout rate of 10%, the target recruitment size was set at 40 participants per group.

As defined in the study protocol, the primary objective was to evaluate whether the mean change in CS-30 score from baseline to 3 months differed between the ERT and placebo groups. Analyses were primarily conducted according on an intention-to-treat (ITT) basis. Additionally, a per-protocol (PP) analysis was performed to assess the effect of ERT under conditions of proper use, excluding participants with protocol deviations. Pairwise deletions were applied to address missing data. For each analysis, only the available data points for relevant variables were included. The baseline score was used as a reference, and the mean changes in scores at 3 and 12 months postintervention were compared between the ERT and placebo groups. Within-group changes and between-group comparisons were analysed using linear regression. Results were reported as means with standard deviations (SDs) for descriptive statistics, while regression analysis outcomes were presented as regression coefficients with 95% CI. Statistical significance was defined as a *P*-value of <.05. No formal correction for multiple testing was applied to the secondary outcomes, which were prespecified in the study protocol. Accordingly, these findings are interpreted as exploratory and hypothesis-generating. In addition, several *post hoc* sensitivity and subgroup analyses were performed. Specifically, the median change from baseline was compared between the groups using the Brunner–Munzel test; the analyses were repeated excluding participants who had undergone TKA; and linear regression models were used to examine the additive effect of ERT on MREP stratified by KL grade and by the median value of baseline knee pain VRS. Furthermore, a multivariable regression analysis was conducted including both KL grade and VRS as explanatory variables to explore their influence on changes in CS-30. Additionally, we conducted a sensitivity analysis using a Poisson regression-based difference-in-differences approach, incorporating group, timepoint and their interaction as fixed effects. As these subgroup and sensitivity analyses were not prespecified in the study protocol, their results should be interpreted with caution as exploratory in nature. All statistical analyses were conducted using SAS V.9.4 TS1M8 (V.9.4M8) by an analyst who was blinded to group assignment, while sensitivity analyses and figure generation were performed using Python (v3.10.12).

## Results

**Figure 1:**
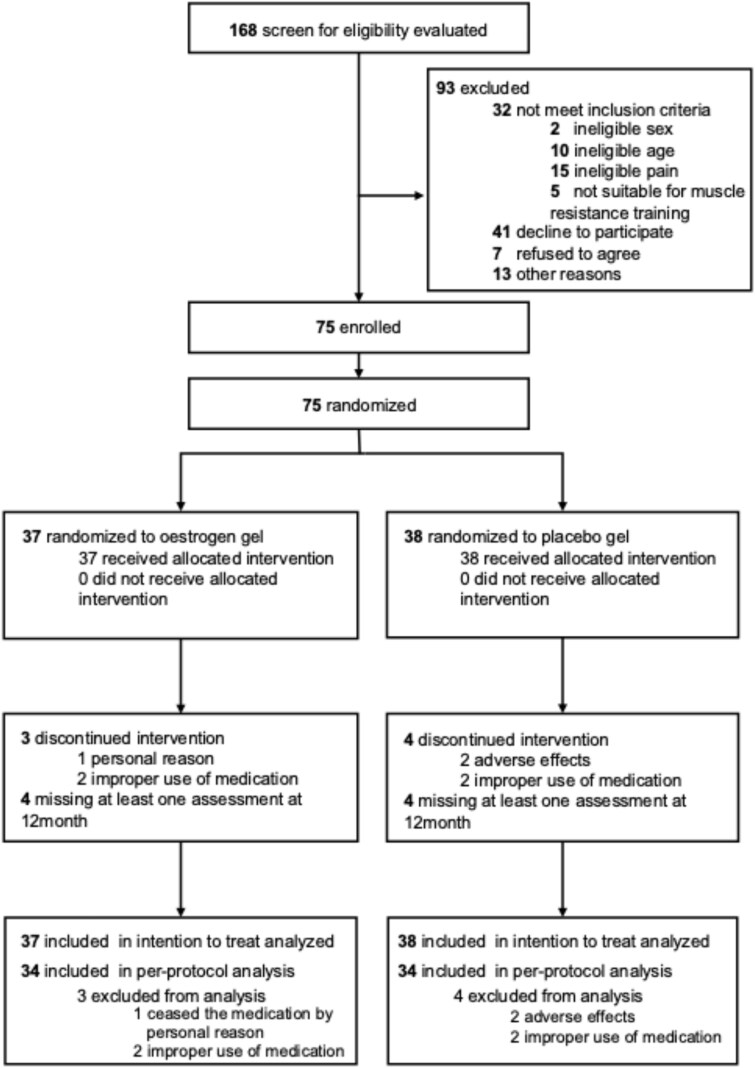
Flow diagram of the study progression.

Recruitment was conducted from 17 December 2021 to 30 October 2024. A total of 168 individuals applied for the study, attended informational sessions and underwent eligibility screenings. Of these 168 individuals, 93 were deemed ineligible and 75 were subsequently enrolled in the study (Figure 1). Final enrolment took place on 23 August 2023. The stratified randomised Group A comprised 37 participants, while Group B included 38 participants. As of August 2024, only three participants had withdrawn from the study, which was significantly lower than the initially anticipated dropout rate of 10%. With a planned sample size of 36 participants per group, registration was successfully completed with 75 participants.

**Table 1:** Baseline characteristics of randomised women (ITT analysis).

	Participants, mean (SD)		
	Total population	ERT	Placebo
	*n* = 75	*n* = 37	*n* = 38
Age, years	73.79 (5.84)	73.86 (5.99)	73.71 (5.77)
Daily steps, steps/day/week	4420 (2590)	4276 (2587)	4560 (2621)
Average KES, N/kg	20.89 (5.91)	20.30 (4.87)	21.46 (6.78)
Baseline			
Physical measurements			
Height, m	1.53 (0.04)	1.54 (0.05)	1.53 (0.03)
Weight, kg	52.95 (8.02)	52.85 (7.15)	53.06 (8.88)
BMI, kg/m^2^	22.55 (3.23)	22.31 (2.52)	22.77 (3.83)
Thigh circumference, cm	44.48 (4.38)	44.62 (4.46)	44.34 (4.35)
Calf circumference, cm	34.08 (2.84)	34.05 (2.93)	34.11 (2.79)
Body composition			
Lower limb muscle mass, kg	12.26 (1.52)	12.35 (1.50)	12.18 (1.55)
Trunk muscle mass, kg	18.61 (0.97)	18.66 (0.91)	18.57 (1.03)
Skeletal muscle mass index, kg/m^2^	6.54 (0.50)	6.56 (0.48)	6.51 (0.52)
Body fat mass, kg	16.74 (6.21)	15.72 (5.05)	17.75 (7.12)
Total body water, kg	26.63 (2.69)	26.59 (2.38)	26.67 (3.01)
Muscle strength			
Grip strength (bilateral average), kgw	21.58 (3.51)	22.21 (3.73)	21.15 (3.13)
Lower limb strength (bilateral average), N/kg	20.33 (5.69)	20.09 (5.01)	20.58 (6.36)
Physical performance			
CS-30, repetitions	15.20 (3.71)	14.81 (3.95)	15.58 (3.48)
5-m walking time, seconds	3.43 (0.76)	3.41 (0.75)	3.44 (0.77)
TUGT, seconds	7.28 (1.49)	7.11 (1.45)	7.44 (1.53)
Blood tests			
Albumin, mg/dL	4.34 (0.25)	4.39 (0.19)	4.29 (0.29)
C-reactive protein, mg/dL	0.16 (0.25)	0.16 (0.25)	0.15 (0.26)
Calcium, mg/dL	9.71 (0.39)	9.72 (0.35)	9.69 (0.43)
25(OH)Vit-D, ng/dL	21.96 (4.64)	22.49 (4.89)	21.44 (4.39)
Insulin-like growth factor-1, ng/ml	78.62 (25.45)	79.95 (24.629)	77.34 (26.49)
Triglyceride, mg/dL	140.43 (69.52)	141.08 (67.68)	139.80 (72.16)
Low density lipoprotein cholesterol, mg/dL	121.71 (25.17)	124.95 (23.76)	118.55 (26.41)
Haemoglobin A1c, %	5.69 (0.55)	5.63 (0.46)	5.75 (0.63)
Questionnaires (pain and health perception)			
SF-12, physical function score	45.87 (11.52)	46.63 (9.93)	45.13 (12.97)
SF-12, mental health score	52.42 (7.92)	50.12 (7.84)	54.66 (7.43)
SF-12, social function score	48.73 (8.57)	48.89 (8.90)	48.57 (8.35)
Visual analogue scale (daily average knee pain)	27.75 (14.05)	28.24 (14.38)	27.26 (13.89)
Knee x-ray (Kellgren and Lawrence classification), no. (%)			
0	0	0	0
1	13 (17)	7 (19)	6 (16)
2	47 (63)	25 (67)	22 (58)
3	12 (16)	5 (14)	7 (18)
4	0	0	0
Total knee replacement	3 (4)	0	3 (8)

The analysis included all 75 participants, whose mean age was 73.8 years (SD 5.8), with a mean body mass index (BMI) of 22.6 (SD 3.2) kg/m^2^ and a CS-30 score of 15.2 (SD 3.7) repetitions (Table 1). After statistical analysis, unblinding revealed that Group A received an oestrogen gel, whereas Group B received a placebo gel. All 75 randomised participants completed at least 75% of the group sessions and home exercises. However, four participants were identified as improperly using the gel, defined as having >15% of the original amount remaining at the end of the intervention. Two participants in the placebo group dropped out due to adverse effects, and one participant in the ERT group discontinued gel application for family reason. The PP analysis included 34 participants in each group (Figure 1). Baseline comparisons were conducted to assess the balance between groups. The groups were similar in demographic characteristics, BMI, muscle strength, muscle mass, physical performance and pain assessment, with no notable disparities observed. The serum oestradiol levels are presented in Table 2; no participants in either group had levels exceeding 20 pg/ml.

**Table 2:** Serum oestradiol levels at baseline, 3, and 12 months in ERT and placebo groups.

Time point	Serum E2 level^a^	ERT (*n* = 37)		Placebo (*n* = 38)	
		*n* (%)	Mean (SD)	*n* (%)	Mean (SD)
Baseline					
	<10 pg/ml	31 (83.8)	–	31 (81.6)	–
	**≥**10 pg/ml	6 (16.2)	13.52 (4.21)	7 (18.4)	12.20 (1.81)
	Missing data	0	–	0	–
3 months					
	<10 pg/ml	2 (5.4)	–	30 (78.9)	–
	**≥**10 pg/ml	35 (94.6)	30.16 (10.01)	8 (21.1)	13.95 (2.91)
	Missing data	0	–	0	–
12 months					
	<10 pg/ml	33 (89.2)	–	32 (84.2)	–
	**≥**10 pg/ml	0	–	2 (5.3)	10.50 (0.42)
	Missing data	4 (10.8)	–	4 (10.5)	–

Abbreviation: E2, oestradiol

^a^Serum oestradiol levels were assessed using venous blood samples obtained at baseline and 3 and 12 months after the start of the intervention. For values below the detection threshold of 10 pg/ml, quantitative measurement was not possible; thus, the number and proportion of participants with undetectable levels are reported. For values equal to or above 10 pg/ml, mean concentrations and SDs are presented.

**Figure 2:**
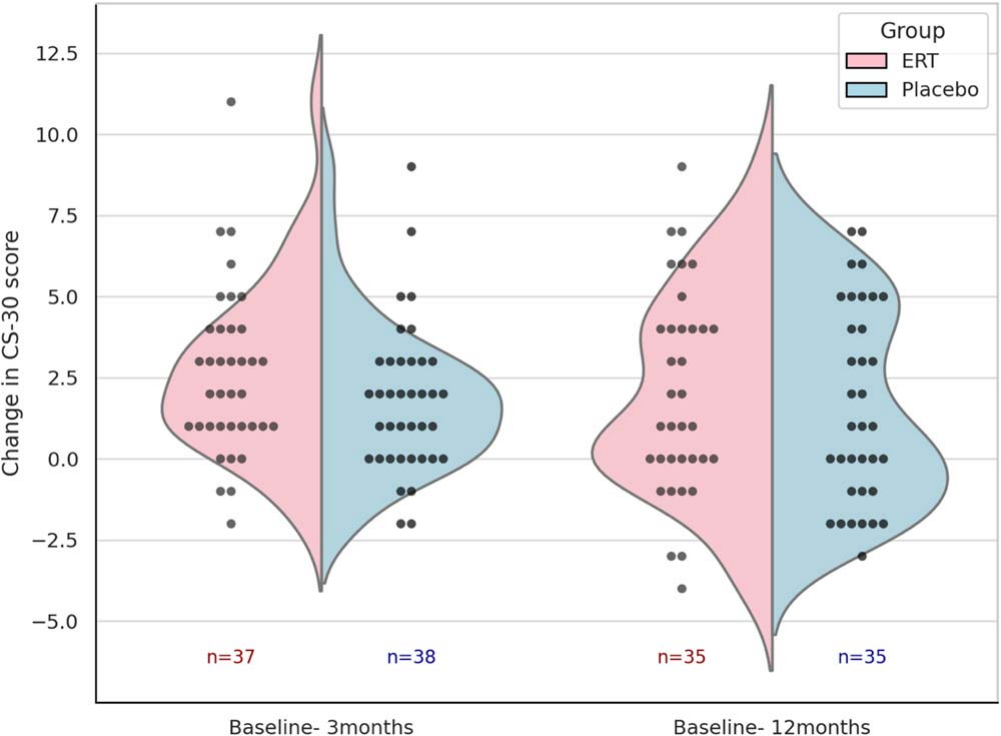
Comparison of changes between the Oestrogen group and the Placebo group at 3 and 12 months postintervention. This figure illustrates the changes in CS-30 scores from baseline to 3 months postintervention and 12 months postintervention (9 months after the end of the intervention), comparing the ERT group (Group = 0) and the placebo group (Group = 1). The distribution of score changes in each group is shown using violin plots, with each half representing one group (left: ERT, right: placebo). These curves visualise the kernel density estimation of the distribution of the score changes. These curves visualise the kernel density estimation of the distribution of the score changes. Overlaid black dots indicate individual data points. The sample size (*n*) for each group is shown within the plot.

The baseline CS-30 score was 14.81 (SD 3.95) repetitions in the ERT group and 15.58 (SD 3.48) repetitions in the placebo group (Table 1). In the ITT analysis, the mean change from baseline at the 3-month, immediately after the intervention, was 2.59 (SD 2.58) in the ERT group and 1.79 (SD 2.28) in the placebo group, as shown in Table 3. The increase in the ERT group was 0.81 repetitions higher than that in the placebo group, representing a 5.5% increase from the mean baseline value in the ERT group. The percentage increase from baseline was 17.5% in the ERT group and 11.5% in the placebo group, with a relative ratio of 1.52 (Table 3). The distribution of change scores in CS-30 at the end of the intervention followed a normal distribution in each group, and the variability in each case was also described (Figure 2). The linear regression analysis presented in Table 3 indicated that the additive effect of ERT on MREP was not statistically significant [coefficient: 0.81 (95% CI: −0.31, 1.92), *P* = .16]. In contrast, the PP analysis showed mean changes of 2.71 (SD 2.66) in the ERT group and 1.53 (SD 1.94) in the placebo group, suggesting a possible additive effect of ERT on MREP [coefficient: 1.18 (95% CI: 0.05, 2.30), *P* = .04]. At 12 months postbaseline, 9 months postintervention, both groups maintained improvements from baseline, but no significant difference was observed between them.

**Table 3:** Comparison of changes in CS-30 scores from baseline to 3 and 12 months postintervention between ERT and placebo groups: results from ITT and PP analyses.

	Mean CS-30 scores and percentage changes at each time point^a^, mean (SD), (%)		CS-30 score change from baseline, repetitions, mean (SD)		Univariable linear regression analysis^b^		
	ERT	Placebo	ERT	Placebo	Coefficient value^b^	95%CI	*P*-value
(i) Baseline to 3 months, immediately after the intervention							
Primary analysis, ITT analysis	17.41 (4.25), 17.52	17.37 (3.85), 11.49	2.59 (2.58)	1.79 (2.28)	0.81	[−0.31, 1.92]	0.16
PP analysis	17.12 (4.28), 18.78	17.03 (3.90), 9.87	2.71 (2.66)	1.53 (1.94)	1.18	[0.05, 2.30]	0.04
(ii) Baseline to 12 months, 9 months postintervention							
Primary analysis, ITT analysis	16.60 (4.83), 12.08	17.20 (4.19), 10.41	2.00 (3.14)	1.63 (3.01)	0.37	[−1.1, 1.84]	0.61
PP analysis	16.47 (4.84), 14.29	17.06 (4.28), 10.07	2.06 (3.17)	1.58 (2.88)	0.69	[−0.79, 2.16]	0.36

^a^The mean (SD) CS-30 scores at each time point (3 and 12 months after intervention) for both the ERT and placebo groups. The percentage change based on group means, derived from the change in group-average scores at each time point relative to baseline.

^b^Placebo serves as the reference for the regression coefficient, and the effect of ERT is compared.

^c^Linear regression analysis was conducted to compare the changes between the intervention and placebo groups. Specifically, changes from baseline to 3 and 12 months postintervention were analysed. The effects of the intervention were evaluated using univariable linear regression analysis, and the results were expressed as regression coefficients, 95% CIs and *P*-values to indicate the differences between groups.

The results for the secondary outcomes from the ITT analysis are presented in Tables 4 and 5, while those from the PP analysis are available in Appendices 4 and 5 in the supplementary data. At 3 months postintervention, no significant differences were found between the groups in anthropometric measures, muscle strength or physical performance (5-m walking time and TUGT) attributable to ERT. Regarding trunk muscle mass, the mean change was −0.15 (SD 0.30) kg in the ERT group and −0.003 (SD 0.24) kg in the placebo group. The regression coefficient was −0.15 [95% CI: −0.27, −0.02], with a *P*-value of .02, indicating a significant reduction in trunk muscle mass in the ERT group. No significant difference was observed in the change in lower limb muscle mass between the two groups. The change in KES was greater in the ERT group (4.27, SD 5.62 N/kg) than in the placebo group (2.56, SD 5.38 N/kg), with a difference of 1.71 N/kg. This corresponded to an 8.4% increase from baseline in the ERT group. Focusing on knee pain and quality of life, the SF-12 mental health score showed a significant increase in the ERT group [coefficient: 8.04 (95% CI: 0.32, 15.76), *P* = .04]. Regarding the VRS, the mean change was −9.22(SD 10.86) in the ERT group and −5.03 (SD 15.92) in the placebo group, indicating a greater reduction in pain scores in the ERT group, although the difference was not statistically significant. At 12 months postintervention, calf circumference was significantly greater in the ERT group (Table 5). Although no other significant differences were observed.

**Table 4:** Changes in secondary outcomes from baseline to 3 months postintervention in ERT and placebo and between group comparison (ITT analysis).

	Score change from baseline to 3 months, mean (SD)		Univariable linear regression analysis^a^		
	ERT (*n* = 37)	Placebo (*n* = 38)	Coefficient value^a^	95%CI	*P*-value
Physical measurements					
BMI, kg/m^2^	0.22 (0.78)	0.12 (0.58)	0.10	[−0.22, 0.41]	0.54
Thigh circumference, cm	−0.42 (2.55)	−0.32 (1.80)	−0.10	[−1.11, 0.91]	0.84
Calf circumference, cm	0.32 (1.75)	−0.09 (1.42)	0.42	[−0.32, 1.15]	0.26
Body composition					
Lower limb muscle mass, kg	−0.06 (0.90)	−0.27 (0.69)	0.21	[−0.17, 0.58]	0.27
Trunk muscle mass, kg	−0.15 (0.30)	−0.003 (0.24)	−0.15	[−0.27, −0.02]	0.02
Body fat mass, kg	0.81 (1.060)	0.48 (1.08)	0.34	[−0.16, 0.83]	0.18
Total body water, kg	−0.43 (0.63)	−0.24 (0.59)	−0.20	[−0.48, 0.08]	0.18
Muscle strength					
Grip strength (bilateral average), kgw	0.52 (1.82)	0.34 (1.46)	0.18	[−0.58, 0.93]	0.64
Lower limb strength (bilateral average), N/kg	4.27 (5.62)	2.56 (5.38)	1.71	[−0.82, 4.24]	0.18
Physical performance					
5-m walking time, seconds	−0.35 (0.72)	−0.07 (1.01)	−0.28	[−0.68, 1.25]	0.17
TUGT, seconds	−0.59 (1.01)	−0.51 (1.30)	0.07	[−0.61, 0.46]	0.79
Blood tests					
Albumin, mg/dL	−0.01 (0.17)	−0.07 (0.22)	0.05	[−0.04, 0.14]	0.26
C-reactive protein, mg/dL	−0.06 (0.25)	−0.02 (0.35)	−0.04	[−0.18, 0.10]	0.56
Calcium, mg/dL	0.01 (0.31)	−0.03 (0.34)	0.06	[−0.11, 0.19]	0.62
25(OH)Vit-D, ng/dL	−1.77 (2.94)	−2.04 (2.92)	−0.27	[−1.09, 1.63]	0.69
Insulin-like growth factor-1, ng/ml	1.36 (11.30)	4.44 (15.18)	−3.08	[−9.31, 3.15]	0.33
Triglyceride, mg/dL	−5.11 (50.08)	−5.69 (64.55)	0.58	[−26.29, 27.46]	0.97
Low density lipoprotein cholesterol, mg/dL	−1.55 (22.74)	0.002 (14.04)	−2.39	[−10.26, 7.15]	0.72
Haemoglobin A1c, %	−0.04 (0.23)	−0.04 (0.21)	0.001	[−0.10, 0.10]	0.99
Questionnaires (pain and health perception)					
SF-12, physical function score	−1.39 (20.65)	7.89 (21.04)	−9.25	[−18.78, 0.29]	0.06
SF-12, mental health score	5.56 (10.54)	−2.63 (21.19)	8.04	[0.32, 15.76]	0.04
SF-12, social function score	5.56 (21.64)	9.21 (21.29)	−3.81	[−13.62, 6.01]	0.44
Visual analogue scale	−9.22 (10.86)	−5.03 (15.92)	−4.19	[−10.48, 2.10]	0.18

^a^Placebo serves as the reference for the regression coefficient, and the effect of ERT is compared.

^b^Linear regression analysis was conducted to compare the changes between the intervention and placebo groups. Specifically, the average values at baseline were compared to those at the first postintervention measurements, and the differences were analysed. The effects of the intervention were evaluated using univariable linear regression analysis, and the results were expressed as regression coefficients, 95% CIs and *P*-values to indicate the differences between groups.

**Table 5:** Changes in secondary outcomes from baseline to 12 months postintervention in ERT and placebo, and between group comparison (ITT analysis)

	Score change from baseline to 3 months, mean (SD)		Univariable linear regression analysis^a^		
	ERT (*n* = 37)	Placebo (*n* = 38)	Coefficient value^b^	95%CI	*P-*value
Physical measurements					
BMI, kg/m^2^	0.25 (0.79)	0.18 (0.64)	0.07	[−0.27, 0.41]	0.69
Thigh circumference, cm	−1.7 (3.59)	−1.86 (3.29)	0.16	[−1.48, 1.8]	0.85
Calf circumference, cm	0.5 (1.34)	−0.24 (1.23)	0.74	[0.13, 1.36]	0.02
Body composition					
Lower limb muscle mass, kg	0.15 (0.94)	−0.23 (0.77)	0.38	[−0.04, 0.79]	0.08
Trunk muscle mass, kg	0.00 (0.25)	0.07 (0.26)	−0.07	[−0.19, 0.05]	0.24
Body fat mass, kg	0.44 (1.68)	0.12 (1.13)	0.32	[−0.36, 1.01]	0.35
Total body water, kg	0.05 (0.75)	0.15 (0.72)	−0.09	[−0.45, 0.25]	0.58
Muscle strength					
Grip strength(bilateral), kgw	−0.22 (1.54)	−0.23 (1.93)	−0.01	[−0.83, 0.84]	0.99
Lower limb strength (bilateral average), N/kg	3.34 (5.15)	2.28 (6.01)	1.07	[−1.60, 3.74]	0.43
Physical performance					
5-m walking time, seconds	−0.15 (0.77)	0.16 (0.79)	−0.31	[−0.68, 0.06]	0.10
TUGT, seconds	−0.28 (1.35)	−0.27 (1.44)	−0.01	[−0.67, 0.66]	0.98
Blood tests					
Albumin, mg/dL	−0.03 (0.21)	0.44 (3.02)	−0.48	[−1.55, 0.59]	0.37
C-reactive protein, mg/dL	−0.03 (0.27)	−0.05 (0.27)	−0.02	[−0.11, 0.15]	0.78
Calcium, mg/dL	0.16 (0.27)	0.14 (0.42)	0.02	[−0.15, 0.2]	0.81
25(OH)Vit-D, ng/dL	−2.55 (3.07)	−2.45 (4.82)	−0.1	[−2.1, −1.9]	0.92
Insulin-like growth factor-1, ng/ml	−1.56 (14.30)	−2.02 (15.98)	0.46	[−7.01, 7.93]	0.90
Triglyceride, mg/dL	13.97 (68.47)	21.52 (59.84)	−7.55	[−39.12, 24.02]	0.63
Low density lipoprotein cholesterol, mg/dL	−1.00 (22.12)	2.68 (13.94)	−3.68	[−12.71, 5.36]	0.42
Haemoglobin A1c, %	0.10 (0.19)	0.08 (0.16)	0.02	[−0.07, 0.35]	0.64
Questionnaires (pain and health perception)					
SF-12, physical function score	−1.43 (25.68)	1.43 (27.08)	−2.86	[−15.45, 9.73]	0.65
SF-12, mental health score	1.43 (15.69)	0.00 (20.78)	1.43	[−7.35, 10.21]	0.74
SF-12, social function score	1.43 (21.81)	5.71 (24.31)	−4.29	[−15.30, 6.73]	0.44
Visual analogue scale	−4.91 (13.90)	0.09 (14.81)	−5.0	[−11.85, 1.85]	0.15

^a^Linear regression analysis was conducted to compare the changes between the intervention and placebo groups. Specifically, the average values at baseline were compared to those at second postintervention measurements, and the differences were analysed. The effects of the intervention were evaluated using univariable linear regression analysis, and the results were expressed as regression coefficients, 95% CIs and *P*-values to indicate the differences between groups.

^b^The regression coefficient represents the comparison between groups, with the placebo group serving as the reference.

Table 6 presents adverse effects associated with the intervention in this study. During the outcome assessment conducted after the intervention, irregular genital bleeding, a typical adverse effect of ERT, was observed in 3 (8.1%) of 37 participants in the ERT group. This occurred after the cessation of the 3-month topical application. Clinical examinations revealed no endometrial thickening, and follow-up was conducted every 3 months in an outpatient setting. No recurrence was observed during the 9 months following the end of the intervention. Breast tenderness and increased vaginal discharge were reported in 3 (8.1%) of 37 participants and increased vaginal discharge in two (5.4%) of the 37 participants, respectively. No abdominal discomfort or nausea was reported in any participant. During the intervention, skin rashes occurred in two (5.3%) of the 38 participants in the placebo group. These rashes resolved promptly after discontinuation of topical application, and no recurrence was observed.

**Table 6:** Summary of adverse events related to ERT and resistance training.

	Total population	ERT group	Placebo group
	*n* = 75	*n* = 37	*n* = 38
ERT related side effects			
Irregular bleeding	4 (5.3%)	3 (8.1%)	1 (2.6%)
Abnormal uterine bleeding	3 (4.0%)	3 (8.1%)	0
Irregular vaginal bleeding	0	0	1 (2.6%)
Breast tenderness	3 (4.0%)	3 (8.1%)	0
Abnormal vaginal discharge	3 (4.0%)	2 (5.4%)	1 (2.6%)
Serious adverse effects	0	0	0
Venous thromboembolism	0	0	0
Stroke	0	0	0
Coronary artery disease	0	0	0
Gallstones	0	0	0
MREP related side effects			
Muscle soreness	13 (17.3%)	5 (13.5%)	8 (21.1%)
Pain exacerbation			
Knee pain	9 (12.0%)	4 (10.8%)	5 (13.2%)
Low back pain	10 (13.3%)	4 (10.8%)	6 (15.8%)
Palpitation	3 (4.0%)	2 (5.4%)	1 (2.6%)
Fatigue	2 (2.7%)	1 (2.7%)	1 (2.6%)
Dizziness	1 (1.3%)	0	1 (2.6%)
Shortness of breath	1 (1.3%)	1 (2.7%)	0
Serious adverse effects	0	0	0
Bone fracture	0	0	0
Tendon strain	0	0	0
Cardiovascular complication	0	0	0
Rhabdomyolysis	0	0	0
Other adverse effects related to this study			
Skin irritation at application site	2 (2.7%)	0	2 (5.3%)

For sensitivity analysis, the median change in scores between the two groups was evaluated (Appendix 6 in the supplementary data). In the PP analysis, a significant difference in the change in the CS-30 scores was identified (ERT: 3.0; placebo: 1.50; *P* = .05). Appendix 7 in the supplementary data presents the results of a similar between-group comparison conducted after excluding TKA cases. No substantial changes were observed in the primary outcome, and no significant differences were found between the two groups. Table 7 shows the changes in CS-30 according to the KL classification for KOA severity. In participants with KL Grade 1, the average change in CS-30 was significantly greater in the ERT group compared with the placebo group [coefficient: 2.05 (95% CI: 0.14, 3.96), *P* = .03]. However, in KL Grade 3, the change tended to be larger in the placebo group. Furthermore, when participants were stratified into two groups based on the median of the weekly average pain VRS, as shown in Appendix 8 in the supplementary data, the ERT group in the high-pain category exhibited a tendency towards a greater improvement in CS-30, although the difference did not reach statistical significance [coefficient: 1.55 (95% CI: −0.16, 3.27), *P* = .07]. A multivariate analysis incorporating KOA severity and VRS was conducted to assess the additive effect of ERT on CS-30 (Appendix 9 in the supplementary data). In the PP analysis, although the effect of ERT on CS-30 was slightly diminished, there was a tendency for the ERT group to exhibit a greater improvement compared with the placebo group [coefficient: 1.14 (95% CI: −0.04, 2.32), *P* = .06]. In addition, a difference-in-differences analysis using Poisson regression showed that the ERT group exhibited an ~5.3% greater improvement in CS-30 scores compared with the placebo group at 3 months (Appendix 10 in the supplementary data); however, this difference was not statistically significant (coefficient: 0.053, 95% CI: −0.106 to 0.212, *P* = .52).

**Table 7:** Comparison of CS-30 score changes before and after intervention between ERT and placebo groups by KOA stage.

KL classification		CS-30 score changes^a^, mean (SD)		Univariate linear regression model^b^		
Grade	Number (%)	ERT group	Placebo group	Regression coefficient ^c^	95%CI	*P*-value
1	13 (17.3)	3.71 (1.60)	1.67 (1.51)	2.05	[0.14, 3.96]	0.03
2	47 (62.7)	2.64 (2.77)	1.82 (2.81)	0.82	[−0.82, 2.46]	0.32
3	12 (16)	0.80 (1.92)	1.86 (1.35)	−1.06	[−3.15, 1.03]	0.29
4	0	–	–	–	–	–
TKA	3 (4)	–	1.67 (1.53)	–	–	–

Abbreviation: KL, Kellgren and Lawrence

^a^The CS-30 score change represents the individual change for each participant from the baseline before the intervention to immediately after the intervention (3 months later).

^b^This univariate analysis is a linear regression analysis with the CS-30 score change as the dependent variable and the intervention group as the independent variable. The ordinary least squares method was used to estimate the regression coefficient, 95% CI and *P*-value.

^c^This regression coefficient represents the effect of the ERT group relative to the placebo group, which serves as the reference category in the model.

## Discussion

This study was the first trial to investigate the additive effects of combining ERT with MREP on physical performance in older women with KOA. Implemented as a double-blind, randomised controlled trial, it included a 3-month intervention period and a 9-month observation period. This design allowed for the exploration of the potential impact of short-term pharmacological intervention on the effectiveness of muscle resistance exercise while also evaluating the safety of ERT in older women. Although the combination of resistance exercise and ERT resulted in an overall increase of 2.6-repetitions in CS-30 scores (a 17.5% improvement from baseline), the additional effect attributable to ERT alone was limited to 0.81 repetitions (5.5%), and this difference was not statistically significant in the primary ITT analysis. Therefore, the findings do not provide robust evidence for an additive benefit of ERT when combined with MREP in this population.

Several factors could explain these findings. First, the variation in baseline CS-30 scores compromised the study’s statistical power, thereby necessitating a larger sample size. The study aimed to detect a 13% additive effect on CS-30 among patients with KOA, but the observed increase was limited to 5.5%. The baseline mean CS-30 in this cohort was 15.2 repetitions—substantially higher than previously reported values of 9.2—which may have restricted the scope for further improvement and consequently reduced the ability to detect a true effect of ERT with the given sample size. While these analyses were exploratory and the excluded cases may have introduced selection bias (Appendix 11 in the supplementary data), the PP and sensitivity analyses suggested greater improvement in CS-30 scores in the ERT group relative to placebo. These findings suggest that future trials focusing on more narrowly defined populations and recalculating sample size based on the effect size observed in the present study may enhance the likelihood of detecting a true treatment effect.

As a second consideration, the low-dose oestrogen regimen (0.54 mg/day) and the 3-month intervention period may have been insufficient to elicit a full therapeutic effect. Previous studies, such as that by Taaffe *et al.* [38], demonstrated a significant effect size (standard mean difference of 0.61) when combining 1–2 mg/day of oral oestrogen with resistance training for 6–12 months in early postmenopausal women [38–40]. In contrast, our study employed a lower-dose transdermal formulation, selected for its favourable pharmacokinetic profile—specifically, the avoidance of hepatic first-pass metabolism and more stable systemic absorption—allowing effective delivery with fewer adverse events, which supports its safety in older women. However, transdermal ERT may produce different pharmacokinetic dynamics compared to oral formulations, potentially affecting tissue-specific responses. Moreover, our study targeted older women with joint disease, a group more susceptible to functional decline. These participants had been postmenopausal for over 10 years, during which oestrogen receptor degeneration could have occurred [41–43]. This reduction, particularly within muscle and intra-articular tissues, may have attenuated the physiological responsiveness to lower-dose ERT. These factors, along with the age-related downregulation of oestrogen receptors, should be taken into account when interpreting the findings. Additionally, the selected dose may have led to an overestimation of the expected effect size in the sample size calculation. Nevertheless, the absence of a significant benefit in this study should not be interpreted as a refutation of ERT’s broader therapeutic potential. Rather, it underscores the importance of optimising dose, route and timing of administration for different target populations.

Thirdly, the observed effect size may have been influenced by participant characteristics, particularly variations in KOA severity and the inclusion of individuals with KL grade 1 to TKA. At the time of study design, a broad inclusion strategy was adopted to reflect the wide applicability of muscle resistance exercise across varying stages of KOA and to enhance generalisability to routine clinical practice [32, 33]. However, this heterogeneity may have diluted treatment effects. A sensitivity analysis stratified by KL grade (Table 7), although exploratory, suggested that ERT could particularly offer benefits in early-stage KOA with limited structural degeneration (KL Grade 1). These exploratory findings could lead to the hypothesis that initiating ERT in combination with MREP before disease progression may help preserve physical function. Future trials targeting more homogeneous populations, such as those with KL Grades 1–2, may allow for a clearer evaluation of treatment efficacy.

Another limitation is the absence of biological assessments, which prevented direct verification of the receptor-mediated mechanisms by which oestrogen is thought to modulate synovial function, pain signalling and muscle regeneration [21, 22, 44–46]. Therefore, such interpretations remain speculative. Moreover, a small but statistically significant reduction in trunk muscle mass was observed in the ERT group. Although the absolute change was minimal and likely not clinically meaningful, it appears inconsistent with oestrogen’s presumed anabolic effects. This discrepancy may reflect several factors—including the lack of trunk-specific resistance training, limitations of bioelectrical impedance analysis in older adults and possible statistical fluctuation—and should therefore be interpreted with caution.

Conversely, the SF-12 mental health score increased in the ERT group, suggesting a potential role of oestrogen in supporting mental well-being in older women with KOA. Oestrogen modulates serotonin and dopamine pathways and attenuates hypothalamic–pituitary–adrenal axis overactivity, mechanisms linked to reduced depression and anxiety [47]. Postmenopausal oestrogen decline is associated with increased mental health risks, and ERT may alleviate psychological stress alongside pain reduction, enhancing quality of life. Notably, the observed improvement in the SF-12 mental health component score (+8.04 points) exceeded the minimal clinically important difference of approximately 6.24 points, corresponding to a 12% increase from baseline as reported in rehabilitation studies of lower extremity osteoarthritis [48, 49]. These findings suggest potential clinical relevance, although the underlying mechanisms remain unclear. Finally, the observed 8.4% increase in knee extension strength in the ERT group, although not statistically significant compared to placebo (*P* = .18), can be noted. Previous trials without resistance training reported gains of 6% [40]; however, direct comparisons are challenging due to differing study designs.

This study also evaluated adverse effects. The Women’s Health Initiative previously reported increased risk of thromboembolism, cardiovascular disease and cerebrovascular events with oral combined HRT in women over 60 [50]. In contrast, recent evidence suggested that low-dose conjugated oestrogens used in oral hormone therapies are associated with a lower risk profile, making clinical studies feasible under carefully managed conditions [51]. In our trials, transdermal administration was used to bypass hepatic metabolism and reduce such risks. Minor adverse events included irregular genital bleeding (8.1%, 3/37), breast tenderness (8.1%, 3/37) and abnormal vaginal discharge (5.4%, 2/37), with no serious events reported. Although participants were older and postmenopausal for over 10 years—outside current HRT initiation guidelines [52]—close monitoring, including baseline and post-treatment ultrasonography, helped ensure safety. Those with abnormal uterine bleeding or endometrial thickening received additional evaluations. While low-dose transdermal ERT was well tolerated under these conditions, further studies are needed to determine its long-term safety and appropriate use in women aged 65 years and older.

Several limitations should be acknowledged. First, some subgroup and sensitivity analyses were exploratory and not prespecified, so their findings should be interpreted with caution. Second, the timing of serum estradiol measurements varied and was not optimised to assess treatment exposure. Third, the intervention was of short duration, used a low dose of oestrogen and lacked biological outcome measures such as tissue biopsies. Most participants had only mild KOA, and their relatively lean body composition may limit generalisability to other populations, including those with higher BMI or of different ethnic backgrounds. Finally, although participants were instructed to avoid additional resistance training, physical activity levels may have varied. Although progestogen administration was not included in this trial, we recognise that in routine clinical settings, endometrial protection is essential when prescribing oestrogen to women with a uterus.

## Conclusion

In summary, this randomised controlled trial involving women aged 65 years and older with KOA did not demonstrate a statistically significant additive effect of ERT on overall physical performance when combined with MREP. The observed 5.5% improvement in CS-30 performance associated with ERT was not significant in the primary analysis and should be interpreted cautiously. However, exploratory subgroup analyses suggested that ERT may have benefits under certain clinical conditions, such as in participants with milder KOA severity or among protocol-adherent participants. These findings are hypothesis-generating and highlight the need for further research in more targeted populations and with optimised dosing regimens.

## Supplementary Material

Supplementary_materials_afaf224

aa-25-1188-File003_afaf224

## Data Availability

De-identified clinical data, as well as treatment response data for the cohort included in this study, will be made available upon request to the corresponding author. Requests must come from legitimate researchers and clinicians affiliated with medical or academic institutions and must be for academic or clinical research purposes. Approval for data sharing will need to be obtained from the consortium and partner institutions for each request.

## Clinical Trial Registration

jRCTs061210062.
