# Supplementary material for: Oestrogen replacement combined with resistance exercise in older women with knee osteoarthritis: a randomised, double-blind, placebo-controlled clinical trial
Source: Age Ageing. 2025 Aug 7;54(8):afaf224. doi: 10.1093/ageing/afaf224 (PMC12342371; doi:10.1093/ageing/afaf224)
Supplement: Supplementary_materials_afaf224 [file supplementary_materials_afaf224.docx]

**Oestrogen Replacement Combined with Resistance Exercise in Older Women with Knee Osteoarthritis: A Randomised, Double-Blind, Placebo-Controlled Clinical Trial**

**Supplementary Data Appendix**

**Contents**

Appendix 1. Full list of inclusion and exclusion criteria….………….…………………………….….................................2

Appendix 2. Flowchart of study implementation and participants' visits to medical centres….............................................4

Appendix 3. The trial design schedule……………….………………………………………...............................................5

Appendix 4. Changes in outcomes from baseline to three months post-intervention in ERT and placebo, and between group comparison (Per-protocol analysis) .………………………..………………………….……………………..……...6

Appendix 5. Changes in outcomes from baseline to 12 months post-intervention in ERT and placebo, and between group comparison (Per-protocol analysis) ………………………………………………………………………………………...8

Appendix 6. Comparison between the two groups using the median change in each outcome measured at baseline, three months, and 12 months…………………………………………………………………………………………………......10

Appendix 7. Changes in outcomes from baseline to three months post-intervention in ERT and placebo, and between-group comparison (ITT analysis excluding TKA cases; sensitivity analysis)……………..…………………...........................…12

Appendix 8. Comparison of CS-30 score changes before and after intervention between ERT and Placebo groups by knee pain scale group.………………………………………………………...………..………………….............................…..14

Appendix 9. Multivariable analysis of the additional effect of ERT on CS-30 score changes……………………..…...…15

Appendix 10. Changes in CS-30 scores and difference-in-differences effects at three and 12 months……………………16

Appendix 11. Changes in outcomes from baseline to three months post-intervention in ERT and placebo, and between group comparison (Cases excluded from the ITT analysis due to failure to meet the allocated adherence criteria)…………………………………………………………………………………………..……………...………….17

**Appendix 1. Full list of inclusion and exclusion criteria**

| Inclusion Criteria | Participants must meet all the following criteria:   1. Provided written informed consent for study participation. 2. Female, aged 65 years or older at the time of consent. 3. Diagnosed with knee osteoarthritis (KOA) and experiencing knee pain for more than three months, as confirmed by:    - A diagnosis from their primary care physician.    - Knee pain reported during recruitment, corroborated by X-ray findings indicating joint space narrowing characteristic of KOA. 4. Able to walk independently.   Rationale for Inclusion   - 1. Ethically appropriate.   2. Appropriate age for evaluating the intervention.   3. Target disease suitable for the intervention.   4. Ability to perform the intervention safely. |
| --- | --- |
| Exclusion Criteria | Participants will be excluded if they meet any of the following criteria:   1. History of cardiovascular conditions:    - Myocardial infarction within the past 2 years.    - Ongoing moderate-to-severe aortic stenosis, acute pericarditis, aortic aneurysm, symptomatic angina, symptomatic valvular disease, untreated arrhythmia, or intermittent claudication.    - Phlebitis or venous thrombosis within the past 2 years.    - Poorly controlled atrial fibrillation. 2. Stroke within the past 2 years. 3. Respiratory issues, including:    - Current use of home oxygen therapy.    - Conditions causing airway obstruction, such as thyroid tumors or metastatic tumors. 4. History of depression or schizophrenia within the past 2 years. 5. Lower limb fractures within the past 2 years or upper limb fractures within the past 6 months. 6. Prolonged immobility (e.g., bedridden for more than 1 week within the past 2 months or for more than 2 weeks within the past 6 months). 7. Difficulty with oral intake. 8. Severe renal impairment (on dialysis). 9. Resting systolic blood pressure >200 mmHg or diastolic blood pressure >100 mmHg. 10. History of gynecologic cancers (endometrial, ovarian, peritoneal, or cervical cancer) within the past 5 years. 11. History of breast cancer within the past 5 years. 12. Severe hepatic impairment. 13. Any condition deemed unsuitable for participation by the principal investigator.   Rationale for Exclusion Criteria 1–8. Safety concerns and potential inability to perform the intervention^28)^. 9–12. Safety concerns related to estrogen application. 13. General safety considerations for participants. |

**Appendix 2. Flowchart of study implementation and participants' visits to medical centres**

**Appendix 3. The trial design schedule**

Abbreviation; BIA, bioelectrical impedance analysis; CS-30, 30-second chair stand test; GS, gait speed; MREP, muscle resistance exercise programme; SF-12, short form-12 health survey; VAS, visual analogue scale

**Appendix 4. Changes in outcomes from baseline to three months post-intervention in ERT and placebo, and between group comparison (Per-protocol analysis)**

|  | | **Score change from baseline to 3months, mean (SD)** | | **Univariable Linear Regression Analysis ^a)^** | | |
| --- | --- | --- | --- | --- | --- | --- |
|  | | **ERT**  **(n=37)** | **Placebo**  **(n=38)** | **coefficient value^b)^** | **95%CI** | **p value** |
| Physical measurements | |  |  |  |  |  |
|  | BMI,kg/m2 | 0.21(0.81) | 0.17(0.55) | 0.03 | [-0.30, 0.37] | 0.84 |
|  | Thigh circumference,cm | -0.60(2.56) | -0.37(1.83) | -0.23 | [-1.31, 0.85] | 0.67 |
|  | Calf circumference,cm | 0.19(1.72) | -0.12(1.31) | 0.31 | [-0.43, 1.05] | 0.41 |
| Body composition | |  |  |  |  |  |
|  | Lower limb muscle mass,kg | -0.06(0.92) | -0.22(0.69) | 0.17 | [-0.23, 0.56] | 0.41 |
|  | Trunk muscle mass,kg | -0.16(0.30) | -0.02(0.24) | -0.14 | [-0.27, -0.01] | 0.04 |
|  | Body fat mass,kg | 0.79(1.08) | 0.54(0.88) | 0.25 | [-0.23, 0.73] | 0.30 |
|  | Total body water,kg | -0.46(0.63) | -0.22(0.58) | -0.25 | [-0.54, 0.05] | 0.10 |
| Muscle strength | |  |  |  |  |  |
|  | Grip strength(bilateral), kgw | 0.45(1.89) | 0.43(1.47) | -0.02 | [-0.80, 0.84] | 0.95 |
|  | Lower limb strength (bilateral average), N/kg | 4.55(5.69) | 3.14(5.31) | 1.41 | [-1.25, 4.07] | 0.30 |
| Physical performance | |  |  |  |  |  |
|  | 5-m walking time,seconds | -0.40(0.71) | 0.01(1.02) | -0.40 | [-0.82, 0.02] | 0.06 |
|  | Timed up and go test,seconds | -0.62(1.03) | -0.43(1.33) | -0.20 | [-0.77, 0.38] | 0.49 |
| Blood tests | |  |  |  |  |  |
|  | Albumin,mg/dL | -0.01(0.17) | -0.08(0.21) | 0.06 | [-0.03, 0.16] | 0.17 |
|  | C-reactive protein,mg/dL | -0.06(0.25) | -0.02(0.36) | -0.04 | [-0.19, 0.11] | 0.57 |
|  | Calcium,mg/dL | 0.01(0.32) | -0.06(0.33) | 0.07 | [-0.08, 0.23] | 0.36 |
|  | 25(OH)Vit-D,ng/dL | -1.77(3.02) | -2.16(2.99) | 0.38 | [-1.07, 1.84] | 0.60 |
|  | Insulin-like growth factor-1, ng/mL | 1.56(11.60) | 4.11(15.71) | -2.55 | [-9.24, 4.14] | 0.45 |
|  | Triglyceride,mg/dL | -2.82(50.2) | -3.56(58.16) | -0.74 | [-25.54, 27.03] | 0.96 |
|  | Low density lipoprotein cholesterol,mg/dL | -1.38(23.20) | 0.91(13.85) | -2.29 | [-11.55, 6.96] | 0.62 |
|  | Hemoglobin A1c, % | -0.04(0.23) | -0.04(0.21) | -0.002 | [-0.11, 0.10] | 0.96 |
| Questionnaires (Pain & Health Perception) | |  |  |  |  |  |
|  | SF-12, Physical function score | 0.00(19.46) | 8.09(21.95) | -8.09 | [-18.13, 1.96] | 0.11 |
|  | SF-12, Mental health score | 5.51(10.74) | -2.94(22.42) | 8.46 | [-0.06, 16.97] | 0.05 |
|  | SF-12, Social function score | 5.88(22.24) | 11.03(21.49) | -5.14 | [-15.74, 5.44] | 0.33 |
|  | Visual analog scale | -8.96(10.98) | -4.34(16.69) | -4.62 | [-11.46, 2.22] | 0.18 |

1. Linear regression analysis was conducted to compare the changes between the intervention and placebo groups. Specifically, the average values at baseline were compared to those at the first post-intervention measurements, and the differences were analyzed. The effects of the intervention were evaluated using univariable linear regression analysis, and the results were expressed as regression coefficients, 95% confidence intervals, and p-values to indicate the differences between groups.
2. Placebo serves as the reference for the regression coefficient, and the effect of ERT is compared.

Abbreviation; BMI, body mass index; CI, confidential interval; ERT, estrogen replacement therapy; SD, standard deviation; SF-12, 12-Item Short-Form Health Survey

**Appendix 5. Changes in outcomes from baseline to 12 months post-intervention in ERT and placebo, and between group comparison (per-protocol analysis)**

|  | | **Score change from baseline to 3months, mean (SD)** | | **Univariable Linear Regression Analysis ^a)^** | | |
| --- | --- | --- | --- | --- | --- | --- |
|  | | **ERT**  **(n=37)** | **Placebo**  **(n=38)** | **coefficient value^b)^** | **95%CI** | **p value** |
| Physical measurements | |  |  |  |  |  |
|  | BMI,kg/m2 | 0.24(0.81) | 0.24(0.58) | -0.04 | [-0.38, 0.31] | 0.84 |
|  | Thigh circumference,cm | -1.87(3.50) | -2.08(3.21) | 0.11 | [-1.53, 1.74] | 0.90 |
|  | Calf circumference,cm | 0.43(1.29) | -0.32(1.22) | 0.96 | [-0.21, 1.70] | 0.01 |
| Body composition | |  |  |  |  |  |
|  | Lower limb muscle mass,kg | 0.17(0.95) | -0.20(0.77) | 0.38 | [-0.05, 0.80] | 0.08 |
|  | Trunk muscle mass,kg | 0.00(0.25) | 0.07(0.26) | -0.07 | [-0.19, 0.06] | 0.26 |
|  | Body fat mass,kg | 0.44(1.68) | 0.12(1.13) | -0.28 | [-1.65, 1.09] | 0.68 |
|  | Total body water,kg | 0.03(0.76) | 0.15(0.71) | -0.14 | [-0.11, 0.07] | 0.46 |
| Muscle strength | |  |  |  |  |  |
|  | Grip strength(bilateral), kgw | -0.22(1.54) | 0.02(2.33) | -0.24 | [-1.21, 0.72] | 0.61 |
|  | Lower limb strength (bilateral average), N/kg | 3.25(5.20) | 2.27(6.00) | 0.98 | [-1.76, 3.72] | 0.48 |
| Physical performance | |  |  |  |  |  |
|  | 5-m walking time,seconds | -0.17(0.77) | 0.19(0.78) | -0.38 | [-0.76, 0.002] | 0.05 |
|  | Timed up and go test,seconds | -0.31(1.36) | -0.19(1.35) | 6.98 | [-6.97, 20.94] | 0.32 |
| Blood tests | |  |  |  |  |  |
|  | Albumin,mg/dL | -0.03(0.21) | 0.46(3.11) | -0.49 | [-1.59, 0.61] | 0.38 |
|  | C-reactive protein,mg/dL | -0.03(0.27) | -0.06(0.28) | -2.11 | [-6.37, 2.16] | 0.33 |
|  | Calcium,mg/dL | 0.16(0.27) | 0.12(0.43) | 0.27 | [-0.14, 0.15] | 0.28 |
|  | 25(OH)Vit-D,ng/dL | -2.55(3.07) | -2.67(4.79) | -0.10 | [-2.10, 1.90] | 0.92 |
|  | Insulin-like growth factor-1, ng/mL | -1.56(14.30) | -2.31(16.39) | 2.87 | [-3.33, 9.07] | 0.36 |
|  | Triglyceride,mg/dL | 13.9768.47 | 22.12(57.60) | -6.02 | [-37.53, 25.48] | 0.70 |
|  | Low density lipoprotein cholesterol,mg/dL | -1.00(22.12) | 3.13(14.25) | -3.86 | [-13.18, 5.47] | 0.41 |
|  | Hemoglobin A1c, % | 0.1(0.19) | 0.08(0.16) | -0.34 | [-1.03, 0.35] | 0.32 |
| Questionnaires (Pain & Health Perception) | |  |  |  |  |  |
|  | SF-12, Physical function score | -1.47(26.07) | 0.0(26.52) | -1.47 | [-14.30, 11.36] | 0.82 |
|  | SF-12, Mental health score | 1.47(15.92) | 0.67(21.54) | 0.80 | [-8.42, 10.02] | 0.86 |
|  | SF-12, Social function score | 1.47(22.14) | 8.33(25.52) | -6.86 | [-18.51, 4.78] | 0.24 |
|  | Visual analog scale | -1.32(9.61) | -2.36(8.48) | 0.02 | [-4.82, 4.87] | 0.99 |

1. Linear regression analysis was conducted to compare the changes between the intervention and placebo groups. Specifically, the average values at baseline were compared to those at second post-intervention measurements, and the differences were analyzed. The effects of the intervention were evaluated using univariable linear regression analysis, and the results were expressed as regression coefficients, 95% confidence intervals, and p-values to indicate the differences between groups.

b) The regression coefficient represents the comparison between groups, with the placebo group serving as the reference.

Abbreviation; BMI, body mass index; CI, confidential interval; ERT, estrogen replacement therapy; SD, standard deviation; SF-12, 12-Item Short-Form Health Survey

**Appendix 6. Comparison between the two groups using the median change in each outcome measured at baseline, three months, and 12 months**

|  | | | **Intention to treat analysis** | | | | | | **Per-protocol analysis** | | | | | |
| --- | --- | --- | --- | --- | --- | --- | --- | --- | --- | --- | --- | --- | --- | --- |
|  | | | **Change in pre- and post-intervention (3 Months - Baseline)** | | | **Change in pre- and post-intervention (12 Months - Baseline)** | | | **Change in pre- and post-intervention (3 Months - Baseline)** | | | **Change in pre- and post-intervention (12 Months - Baseline)** | | |
|  | | | **ERT**  **(n=37)** | **Placebo**  **(n=38)** | **p-value ^a)^** | **ERT**  **(n=37)** | **Placebo**  **(n=38)** | **p-value ^a)^** | **ERT**  **(n=34)** | **Placebo**  **(n=34)** | **p-value ^a)^** | **ERT**  **(n=34)** | **Placebo**  **(n=34)** | **p-value ^a)^** |
| Primary outcome | | | | | | | | | | | | | | |
|  | 30-second chair stand test, median, repetitions | | 2.00 | 2.00 | 0.14 | 1.00 | 1.00 | 0.40 | 3.00 | 1.50 | 0.05 | 1.50 | 1.00 | 0.34 |
| Secondary outcome | | | | | | | | | | | | | | |
|  | Physical measurements | |  |  |  |  |  |  |  |  |  |  |  |  |
|  |  | BMI, median, kg/m2 | 0.20 | 0.09 | 0.67 | 0.23 | 0.14 | 0.94 | 0.17 | 0.09 | 0.80 | 0.20 | 0.22 | 0.75 |
|  |  | Thigh circumference, median, cm | -0.50 | -0.25 | 0.98 | -1.50 | -1.00 | 0.81 | -0.50 | -0.25 | 0.75 | -1.50 | -1.00 | 0.84 |
|  |  | Calf circumference, median, cm | 0.00 | 0.00 | 0.24 | 0.00 | -0.50 | 0.01 | 0.00 | 0.00 | 0.32 | 0.00 | -0.50 | 0.01 |
|  | Body composition | |  |  |  |  |  |  |  |  |  |  |  |  |
|  |  | Lower limb muscle mass, median, kg | -0.15 | -0.10 | 0.91 | 0.09 | 0.00 | 0.15 | -0.15 | -0.10 | 0.77 | 0.09 | 0.00 | 0.16 |
|  |  | Trunk muscle mass, median, kg | -0.10 | 0.00 | 0.05 | 0.00 | 0.09 | 0.21 | -0.10 | 0.00 | 0.06 | 0.00 | 0.09 | 0.25 |
|  |  | Body fat mass, median, kg | 0.65 | 0.50 | 0.32 | 0.45 | 0.10 | 0.39 | 0.60 | 0.50 | 0.40 | 0.40 | 0.20 | 0.64 |
|  |  | Total body water, median, kg | -0.40 | 0.00 | 0.15 | 0.00 | 0.19 | 0.46 | -0.55 | 0.00 | 0.08 | 0.00 | 0.19 | 0.34 |
|  | Muscle strength | |  |  |  |  |  |  |  |  |  |  |  |  |
|  |  | Grip strength (bilateral average), median, kgw | 0.75 | 0.10 | 0.44 | -0.15 | -0.50 | 0.71 | 0.52 | 0.17 | 0.83 | -0.15 | -0.39 | 0.82 |
|  |  | Lower limb strength (bilateral average), median, N/kg | 4.25 | 2.60 | 0.23 | 3.70 | 2.30 | 0.74 | 4.37 | 3.15 | 0.38 | 3.05 | 2.30 | 1 |
|  | Physical performance | |  |  |  |  |  |  |  |  |  |  |  |  |
|  |  | Five metre walking time, median, seconds | -0.20 | -0.25 | 0.43 | -0.10 | 0.20 | 0.16 | -0.25 | -0.20 | 0.16 | -0.15 | 0.20 | 0.10 |
|  |  | Timed up and go test, median, seconds | -0.50 | -0.70 | 0.83 | -0.20 | -0.09 | 0.76 | -0.5 | -0.70 | 0.76 | -0.25 | -0.09 | 0.96 |
|  | Questionnaires (Pain & Health Perception) | |  |  |  |  |  |  |  |  |  |  |  |  |
|  |  | SF-12, Physical function score | 0.00 | 0.00 | 0.04 | 0.00 | 0.00 | 0.79 | 0.00 | 0.00 | 0.08 | 0.00 | 0.00 | 0.78 |
|  |  | SF-12, Mental health score | 0.00 | 0.00 | 0.06 | 0.00 | 0.00 | 0.56 | 0.00 | 0.00 | 0.09 | 0.00 | 0.00 | 0.48 |
|  |  | SF-12, Social function score | 0.00 | 0.00 | 0.20 | 0.00 | 0.00 | 0.27 | 0.00 | 0.00 | 0.13 | 0.00 | 0.00 | 0.26 |
|  |  | Visual analog scale (weekly average knee pain), median | -8.50 | -7.00 | 0.38 | -5.50 | -4.50 | 0.27 | -8 | -6.5 | 0.33 | -5.75 | -4 | 0.12 |

1. The Brunner-Munzel test was used to compare the medians between the two groups.

Abbreviation; BMI, body mass index; ERT, estrogen replacement therapy; SD, standard deviation; SF-12, 12-Item Short-Form Health Survey

**Appendix 7. Changes in outcomes from baseline to three months post-intervention in ERT and placebo, and between-group comparison (ITT analysis excluding TKA cases; sensitivity analysis)**

|  | | | **Score change from baseline to 12months, mean (SD)** | | **Univariable Linear Regression Analysis a)** | | |
| --- | --- | --- | --- | --- | --- | --- | --- |
|  | | | **HRT (n=37)** | **Placebo (n=35)** | **coefficient value ^b)^** | **95%CI** | **p value** |
| Primary outcome | | | | | | | |
|  | 30-s chair stand test,repetitions | | 2.59(2.58) | 1.80(2.35) | 0.79 | [-0.37, 1.96] | 0.18 |
| Secondary outcome | | | | | | | |
|  | Physical measurements | |  |  |  |  |  |
|  |  | BMI,kg/m2 | 0.22(0.78) | 0.11(0.59) | 0.10 | [-0.22, 0.43] | 0.52 |
|  |  | Thigh circumference,cm | -0.42(2.55) | -0.35(1.79) | -0.13 | [-1.12, 0.97] | 0.89 |
|  |  | Calf circumference,cm | 0.32(1.75) | 0.01(1.38) | 0.31 | [-0.43, 1.05] | 0.41 |
|  | Body composition | |  |  |  |  |  |
|  |  | Lower limb muscle mass,kg | -0.06(0.90) | -0.26(0.70) | 0.20 | [-0.18, 0.59] | 0.30 |
|  |  | Trunk muscle mass,kg | -0.15(0.30) | -0.002(0.25) | -0.15 | [-0.28, -0.02] | 0.02 |
|  |  | Body fat mass,kg | 0.81(1.06) | 0.51(1.08) | 0.30 | [-0.21, 0.81] | 0.24 |
|  |  | Total body water,kg | -0.43(0.63) | -0.22(0.60) | -0.21 | [-0.51, 0.08] | 0.15 |
|  | Muscle strength | |  |  |  |  |  |
|  |  | Grip strength(bilateral), kgw | 0.52(1.82) | 0.16(1.29) | 0.36 | [-0.39, 1.10] | 0.34 |
|  |  | Lower limb strength (bilateral average), N/kg | 4.27(5.62) | 2.55(5.55) | 1.72 | [-0.91, 4.35] | 0.20 |
|  | Physical performance | |  |  |  |  |  |
|  |  | 5-m walking time,seconds | -0.35(0.72) | -0.06(1.03) | -0.28 | [-0.70, 0.13] | 0.18 |
|  |  | Timed up and go test,seconds | -0.59(1.01) | -0.46(1.33) | -0.12 | [-0.67, 0.43] | 0.43 |
|  | Questionnaires (Pain & Health Perception) | |  |  |  |  |  |
|  |  | SF-12, Physical function score | -1.39(20.65) | 6.43(20.42) | -7.78 | [-17.37, 1.81] | 0.11 |
|  |  | SF-12, Mental health score | 5.56(10.54) | -2.86(22.09) | 8.26 | [0.21, 16.31] | 0.04 |
|  |  | SF-12, Social function score | 5.56(21.64) | 8.57(21.81) | -3.17 | [-13.31, 6.98] | 0.54 |
|  |  | Visual analog scale | -9.22(10.86) | -5.94(15.69) | -3.27 | [-9.59, 3.04] | 0.30 |

a) Linear regression analysis was conducted to compare the changes between the intervention and placebo groups. Specifically, the average values at baseline were compared to those at the 3-month post-intervention measurements, and the differences were analyzed. The effects of the intervention were evaluated using univariable linear regression analysis, and the results were expressed as regression coefficients, 95% confidence intervals, and p-values to indicate the differences between groups.

b) The regression coefficient represents the comparison between groups, with the placebo group serving as the reference.

Abbreviation: BMI, body mass index; ERT, estrogen replacement therapy; SD, standard deviation; SF-12, 12-Item Short-Form Health Survey.

**Appendix 8. Comparison of CS-30 score changes before and after intervention between ERT and Placebo groups by knee pain scale group.**

| **VRS group category ^a)^** | | **CS-30 Score Changes ^b)^, mean (SD)** | | **Univariate Linear regression model ^c)^** | | |
| --- | --- | --- | --- | --- | --- | --- |
| **Classification** | **Number (%)** | **ERT group** | **Placebo group** | **Regression Coefficient ^d)^** | **95% CI** | **p-value** |
| VRS <25 | 36(48) | 2.11(2.03) | 2.11(2.32) | 0.00 | [-1.48, 1.48] | 1.00 |
| VRS ≧ 25 | 39(52) | 3.05(2.99) | 1.5(2.26) | 1.55 | [-0.16, 3.27] | 0.07 |

a) The VRS subgroup was classified based on the median value of 25 for the overall participants. The average knee pain over the past week was scored on a scale from 0–10 cm visual scale, with the score recorded in millimetres, with participants categorized into two groups: those with a score below 25 and those with a score of 25 or higher.

b) The CS-30 score change represents the individual change for each participant from the baseline before the intervention to immediately after the intervention (3 months later).

c) This univariate analysis is a linear regression analysis with the CS-30 score change as the dependent variable and the intervention group as the independent variable. The ordinary least squares method was used to estimate the regression coefficient, 95% confidence interval, and p-value.

d) This regression coefficient represents the effect of the ERT group relative to the placebo group, which serves as the reference category in the model.

Abbreviation; CI, confidence interval; CS-30, 30 second chair stand test; ERT, oestrogen replacement therapy; SD, standard deviation; VRS, visual rating scale

**Appendix 9. Multivariable analysis of the additional effect of ERT on CS-30 score changes**

| Variable ^a)^ | Intention to treat analysis | | | Per protocol analysis | | |
| --- | --- | --- | --- | --- | --- | --- |
|  | Coefficient | 95% CI | P-value | Coefficient | 95% CI | P-value |
| Intervention group ^b)^  (ERT, ref; Placebo) | 0.68 | [-0.47, 1.83] | 0.24 | 1.14 | [-0.04, 2.32] | 0.06 |
| KL classification ^c)^  (grade1 to 3) | -0.41 | [-1.15, 0.37] | 0.27 | -0.63 | [-1.64, 0.37] | 0.21 |
| Knee pain VRS ^d)^  (ref.; <25 group, ≧25,) | 0.37 | [-0.82, 1.56] | 0.54 | 0.29 | [-0.93, 1.51] | 0.63 |

a) A multivariable linear regression analysis was conducted to compare the outcome of CS-30 score changes between the ERT and placebo groups. The model was adjusted for group assignment, KL grade, and knee pain VRS.

b) This regression coefficient represents the effect of the ERT group relative to the placebo group, which serves as the reference category in the model.

c) The KL classification for KOA severity was categorised into grades 1 to 3. There were no participants with grade 4, and cases involving TKA were excluded from the analysis.

d) Participants marked their average knee pain over the past week on a 0–10 cm visual scale, with the score recorded in millimetres. Based on the baseline median value of 25 for the entire cohort, participants were classified into a low and high pain group. The regression coefficient uses the low VRS group as the reference category.

Abbreviation; CI, confidence interval; CS-30, 30 second chair stand test; ERT, oestrogen replacement therapy; KL, Kellgren Lawrence; SD, standard deviation; TKA, total knee arthroplasty; VRS, visual rating scale

**Appendix 10.** **Changes in CS-30 scores and difference-in-differences effects at three and 12 months**

|  | **Time point difference (baseline)** | | | **Difference-in-difference effect ^a)^**  **(Group*Time interaction)** | | |
| --- | --- | --- | --- | --- | --- | --- |
|  | **coefficient estimat^b)^** | **95%CI** | **p value** | **coefficient estimat^c)^** | **95%CI** | **p value** |
| 1. Baseline to three months, immediately after the intervention | | | | | | |
| Primary analysis, intention to treat analysis | 0.109 | [-0.002, 0.220] | 0.055 | 0.053 | [-0.106, 0.212] | 0.52 |
| Per protocol analysis | 0.094 | [-0.024, 0.212] | 0.12 | 0.078 | [-0.090, 0.246] | 0.36 |
| 1. Baseline to 12 months, nine months post-intervention | | | | | | |
| Primary analysis, intention to treat analysis | 0.049 | [-0.007, 0.106] | 0.09 | 0.008 | [-0.074, 0.089] | 0.86 |
| Per protocol analysis | 0.048 | [-0.011, 0.107] | 0.11 | 0.019 | [-0.066, 0.104] | 0.66 |

1. A difference-in-differences (DiD) analysis was conducted to evaluate the additional effect of ERT beyond the muscle resistance exercise program. Specifically, changes in outcome scores from baseline to three months were compared between the ERT and placebo groups. The DiD effect corresponds to the coefficient of the interaction term between group assignment and timepoint in the Poisson regression model. As Poisson regression uses a log link function, the coefficients are expressed on a logarithmic scale and represent relative changes. Estimates and p-values were reported to assess statistical significance.
2. The baseline serves as the reference for the regression coefficient, and the relationship with the scores at three months is evaluated.
3. Placebo serves as the reference for the regression coefficient, and the effect of ERT is compared.

Abbreviation; CI, confidential interval; CS-30, 30 second chair stand test; DiD, difference in difference; ERT, estrogen replacement therapy; SD, standard deviation

**Appendix 11. Changes in outcomes from baseline to three months post-intervention in ERT and placebo, and between group comparison (Cases excluded from the ITT analysis due to failure to meet the allocated adherence criteria)**

|  | | | **Score change from baseline to 3months, mean (SD)** | | **Welch’s t -test^a)^** |
| --- | --- | --- | --- | --- | --- |
|  | | | **HRT (n=3)** | **Placebo (n=4)** | **p-value** |
| Primary outcome | | | | | |
|  | 30-s chair stand test,repetitions | | 1.33(0.57) | 4.00(3.91) | 0.30 |
| Secondary outcome | | | | | |
|  | Physical measurements | |  |  |  |
|  |  | BMI,kg/m2 | 0.30(0.42) | -0.36(0.74) | 0.23 |
|  |  | Thigh circumference,cm | 1.67(1.25) | 0.13(1.70) | 0.25 |
|  |  | Calf circumference,cm | 1.83(1.52) | -0.24(1.23) | 0.34 |
|  | Body composition | |  |  |  |
|  |  | Lower limb muscle mass,kg | -0.15(0.07) | -0.65(0.62) | 0.35 |
|  |  | Trunk muscle mass,kg | 0.05(0.07) | 0.15(0.26) | 0.64 |
|  |  | Body fat mass,kg | 1.30(0.84) | 0.00(2.31) | 0.50 |
|  |  | Total body water,kg | 0.05(0.49) | -0.43(0.73) | 0.47 |
|  | Muscle strength | |  |  |  |
|  |  | Grip strength(bilateral), kgw | 1.28(0.48) | -0.39(1.34) | 0.10 |
|  |  | Lower limb strength (bilateral average), N/kg | 1.18(4.48) | -2.31(3.29) | 0.28 |
|  | Physical performance | |  |  |  |
|  |  | 5-m walking time,seconds | 0.17(0.47) | -0.75(0.62) | 0.09 |
|  |  | Timed up and go test,seconds | -0.07(0.23) | -1.2(0.91) | 0.10 |
|  | Blood tests | |  |  |  |
|  |  | Albumin,mg/dL | -0.05(0.07) | 0.03(0.32) | 0.77 |
|  |  | C-reactive protein,mg/dL | -0.05(0.18) | -0.03(0.2) | 0.94 |
|  |  | Calcium,mg/dL | 0.05(0.07) | 0.25(0.24) | 0.17 |
|  |  | 25(OH)Vit-D,ng/dL | -1.70(0.85) | -1.05(2.31) | 0.73 |
|  |  | Insulin-like growth factor-1, ng/mL | -2.00(2.83) | 7.25(10.66) | 0.32 |
|  |  | Triglyceride,mg/dL | -44.00(41.01) | -23.75(117.01) | 0.83 |
|  |  | Low density lipoprotein cholesterol,mg/dL | -4.50(17.68) | -7.75(15.2) | 0.82 |
|  |  | Hemoglobin A1c, % | 0.00(0.14) | -0.05(0.26) | 0.82 |
|  | Questionnaires (Pain & Health Perception) | |  |  |  |
|  |  | SF-12, Physical function score | -16.67(28.87) | 6.25(12.05) | 0.21 |
|  |  | SF-12, Mental health score | 4.17(7.22) | 0.00(0) | 0.29 |
|  |  | SF-12, Social function score | 0(0) | -6.25(12.5) | 0.44 |
|  |  | Visual analog scale | -12.17(10.97) | -10.86(3.59) | 0.83 |

- - 1. Welch’s t-test was used to compare the mean values between the intervention and placebo groups. Specifically, the average baseline values were compared with those obtained at second post-intervention measurements, and the differences were analysed. The effects of the intervention were evaluated using Welch’s t-test, with the results expressed as mean differences and p-values to indicate between-group differences.

Abbreviation; BMI, body mass index; ERT, estrogen replacement therapy; SD, standard deviation; SF-12, 12-Item Short-Form Health Survey
